# Examination of Different Sporidium Numbers of *Ustilago maydis* Infection on Two Hungarian Sweet Corn Hybrids’ Characteristics at Vegetative and Generative Stages

**DOI:** 10.3390/life13020433

**Published:** 2023-02-03

**Authors:** Lóránt Szőke, Makoena Joyce Moloi, Dávid Kaczur, László Radócz, Brigitta Tóth

**Affiliations:** 1 Institute of Food Science, University of Debrecen, 138 Böszörményi St., 4032 Debrecen, Hungary; 2 Department of Plant Pathology, Faculty of Agriculture, University of Zagreb, 10000 Zagreb, Croatia; 3 Department of Plant Sciences, University of the Free State-Main Campus, P.O. Box 339, Bloemfontein 9300, South Africa; 4 Institute of Plant Protection, University of Debrecen, 138 Böszörményi St., 4032 Debrecen, Hungary

**Keywords:** antioxidant enzymes, chlorophylls, corn smut, fungus infection, maize, MDA, proline, quality

## Abstract

Corn smut is one of the major diseases in corn production. The cob infection causes high economic and quality loss. This research investigated the effects of three different concentrations of corn smut infection (2500, 5000, and 10,000 sporidia/mL) on two Hungarian sweet corn hybrids (Desszert 73 and Noa). Plants were infected at the vegetative (V4–V5) and the generative (V7) stages. The effects of the corn smut infection were evaluated at 7 and 14 days after the pathogen infection (DAPI) at vegetative and at 21 DAPI at generative stages. The photosynthetic pigments (relative chlorophyll, chlorophyll-*a* and *b*, and carotenoids), malondialdehyde (MDA), and proline concentration, activities of the antioxidant enzymes [ascorbate peroxidase (APX), guaiacol peroxidase (POX), and superoxide dismutase (SOD)], morphological characteristics (plant height, stem and cob diameter, cob length, cob and kernel weights), mineral contents (Al, B, Ca, Cr, Cu, Fe, K, Mg, Mn, Na, P, Pb, S, Sr, and Zn), and quality parameters (dry matter, fiber, fat, ash, nitrogen, and protein) were measured. At both sampling times (7 and 14 DAPI) in both hybrids, the corn smut infection reduced the photosynthetic pigments (relative chlorophyll, chlorophylls-*a*, and *b*, and carotenoids) irrespective of the spore concentration. Under the same conditions, the MDA and proline contents, as well as the activities of APX, POX, and SOD increased at both sampling times. The negative effects of the corn smut infection were also observed at the generative stage. Only the 10,000 sporidia/mL of corn smut caused symptoms (tumor growth) on the cobs of both hybrids at 21 DAPI. Similarly, this treatment impacted adversely the cob characteristics (reduced cob length, kernel weight, and 100 grains fresh and dry weight) for both hybrids. In addition, crude fat and protein content, Mg, and Mn concentration of grains also decreased in both hybrids while the concentration of Al and Ca increased. Based on these results, the sweet corn hybrids were more susceptible to corn smut at the vegetative stage than at the generative stage.

## 1. Introduction

Successful crop production depends on many factors, such as crop type [1], variety [2], and environmental factors [3], including climatic conditions [4], soil [5], and water [6]. Biotic and abiotic stresses also affect yield [7]. Direct protection against abiotic factors such as drought [8], salinity [9], extreme cold or heat [10], heavy metal stress [11], and water deficiency or excess [12] is achieved through resistance. However, biotic stresses are also important [13]. The use of pesticides protects against diseases, pests, and weeds (which cause great economic losses) [14,15]. Their use is disadvantageous because it contributes to environmental pollution [16]. In addition, resistance breeding is widely used to reduce the use of pesticides. Plants have a specific defense system that is activated after infection [17]. The cuticle is the plants’ first defense against fungal invasion [18]. The structure of the cuticle can be different in different plant species [19] and its effectiveness against pathogens is also different [20]. When the fungal invasion is successful, plants produce secondary metabolites to protect themselves [21]. Phenolic terpenes and nitrogen/sulfur-containing compounds are synthesized in plants [22]. The roles of phenolic compounds against pathogens are well studied [23]. For example, benzaldehyde (against *Botrytis cinerea*, [24]), protocatechuic acid (against *Colletotrichum circinans*, [25]), salicylic acid (against *Eutypa lata*, [26]), vanillic acid (against *Phytophthora infestans*, [27]), chlorogenic acid (against *Fusarium osysporum*, [28]), naringin (against *Penicillium digitatum*, [29]), flavones (against *Aspergillus*, [30]), oleuropein (against *Phytophthora*, [31]), Nobiletin (against *Phoma tracheophyta*, [32]), Geinstein (*Monilinia fructicola*, [33]), and Hordatin A (against *Helminthosporium sativum*, [34]). Other important molecules in the plant-pathogen interaction are the reactive oxygen species (ROS) [35]. After infection, plant cells produce ROS such as hydrogen peroxide (H_2_O_2_), singlet oxygen (^1^O_2_), superoxide anions (O_2_^−^), and hydroxyl radicals (^−^OH) [36]. ROS can be important molecules for signal transduction (at low concentration) or toxic (at high concentration) for plants [37]. One of the most detrimental effects of ROS is the induction of lipid peroxidation in the cell membrane [38]. ROS are mainly produced in peroxisomes [39], mitochondria [40], and chloroplasts [41]), which can interfere with metabolic processes. To eliminate ROS, plants use antioxidant enzymes [42]. Ascorbate peroxidase (APX) degrades H_2_O_2_ to water using ascorbic acid as a substrate. Several APX isoforms in plants are distributed in different cellular compartments such as chloroplasts, mitochondria, peroxisomes, and cytosol [43]. Superoxide dismutase (SOD) catalyzes the dismutation of the superoxide anion radical (O_2_^−^) to water and H_2_O_2_. Their classification is based on their subcellular location and bound metal cofactor (Cu/Zn, Mn, Fe, and Ni) [44]. Guaiacol peroxidase (POX) is essential for lignin biosynthesis and neutralizes H_2_O_2_ [45]. Corn smut is a major corn pathogen capable of infecting corn at vegetative and generative stages, leading to serious yield losses [46], especially in sweet corn, which has a great economic impact [47]. Corn smut is a biotrophic pathogen, which causes galls on all aerial parts of its host plants but does not cause the death of cells. To evaluate the impact of a biotrophic pathogen on its host plants, many physiological, morphological, biochemical, and quality parameters give an understanding of the impacts of pathogen infection. Biotrophic pathogens live and complete their life cycle in the host plants. To survive, they derive nutrients from the host plant, leading to reduced growth [48]. Corn smut infection has a significant impact on the leaf chlorophyll content, which can be measured using different methods. During corn smut infection, chlorosis would appear 3–5 days after infection, which is an indication of chlorophyll loss [49]. Therefore, measurements of chlorophyll content are valuable. Infection may also cause oxidative stress in plants [50,51]. The oxidative burst in host plants may activate the antioxidative mechanisms. Szőke et al. [52] showed that the corn smut infection increased the activities of antioxidant enzymes (SOD, APX, and POX) and the malondialdehyde (MDA) content in the infected fodder and sweet corn hybrids. 

Corn smut also has a significant impact on sweet corn yield. Clough et al. [53] found a strong correlation between the intensity of the corn smut infection and kernel characteristics. They stated that when the gall size was bigger, fresh weight, length, diameter, and kernel depth were smaller. According to Pál-Fám et al. [54], corn smut infection significantly decreased the ear, grain and cob weights of the fodder corn, causing significant economic damage. Moreover, they stated that the corn smut-infected cob had lower dry matter, fiber, and ash contents. Keszthelyi et al. [55] also stated that the corn smut infection reduced the dry matter, protein, fat, fiber, and ash contents of the fodder corn. 

The effects of different diseases on the nutrient content of the host plant have also been reported. A high concentration of the corn smut infection increased the amounts of Fe and Zn in the shoots and roots of infected plants [56]. The *Candidatus phytoplasma* L. asiaticus infection decreased the N and P contents of citrus species [57]. The Fusarium-infected tomato plants had a lower Cu content compared to uninfected control plants [58]. Mineral nutrients have an important function in the interaction between plants and pathogens [59]. The element contents of crop plants may differ depending on the host plant and the type of plant pathogen [60].

Szőke et al. [52] showed that infection of sweet corn at the vegetative stage with a high amount of corn smut sporidia (10,000 sporidia/mL), under controlled conditions in the greenhouse negatively impacted the photosynthesis pigments and growth parameters. They suggested a follow-up study to establish the effects of corn smut at lower loadings. During the experiment, the two infection periods were simulated that are the most typical in corn cultivation. The first peak of infection mostly affects young corn plants, which are most characteristic during the period of development of mechanical damage caused by the frit fly or mechanical inter-row weeding cultivation. Another such period is the stage of the emergence of young corn cob initiation. The different sporidia concentrations are a good representation of the level of infection pressure, which may be related to the cultivation variants. The physiological changes that occur as a result of the infection are a good representation of the defense reactions and physiological changes of the individual corn plants. These changes in plant physiology are not only correlated with the amount of infectious sporidia material but also strongly depend on the type of crop (for example, sweet or fodder corn) and its phyto-phenological state of development. Therefore, the current research examined the effects of different concentrations (2500, 5000, and 10,000 sporidia/mL) of corn smut inoculum, on different morphological (plant height and stem diameter) and biochemical parameters (chlorophyll, protein, and MDA contents; the activities of SOD, APX, and POX) at the vegetative (V4–V5) and generative (V7) stages. The corn smut attacks the embryonic tissue when the tissue is already in the differentiation phase and smut is not able to infect during this stage, meaning that there will be no tumor formation. 

The goal for including different infection times, which was not established by Szőke et al. [52], was to examine if there are any other roles of corn smut infection besides tumor formation (e.g., effects on quality and quantity). In addition, monosporidial inoculation does not cause tumor formation but has negative impacts on plant growth and several other physiological processes. At the V7 stage, measurements focused on cob parameters like cob length and diameter, kernel weight, 100 grains fresh and dry weight, element content of grains, and the quality characteristics (dry matter, fiber, fat, ash, nitrogen, and protein). The first goal of this study was to examine the tumor formation at the V4–V5 phenological stage because the first hypothesis was that there is no tumor formation at low (2500 sporidia/mL) inoculation, The second hypothesis of this research was that the corn smut infection negatively affects the morphological, physiological, biochemical, and quality parameters, as well as the element content and other quality characteristics of grains irrespective of the lower dosage. The third hypothesis was that the 10,000 sporidia/mL has more negative impacts on the measured parameters relative to the 2500 and 5000 sporidia/mL treatments. Furthermore, the goal was to examine which phenological stage (V4–5 or V7) is more susceptible to the corn smut infection.

## 2. Materials and Methods

### 2.1. Experimental Conditions

**Figure 1:**
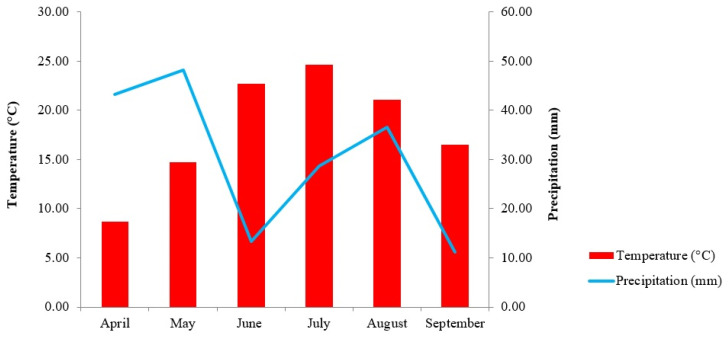
Temperature (°C) and precipitation (mm) during the experimental period. The data originates from the Institute of Plant Protection, the University of Debrecen’s meteorological station (47°33′07.7″ N 21°36′00.3″ E).

The experiment was conducted under field conditions in the Demonstration Garden of the Institute of Plant Protection (47°33′07.7″ N 21°36′00.3″ E). The maize plants [Desszert 73 (Topcorn Ltd., Budapest, Hungary) and Noa (Rédei Kertimag Ltd., Réde, Hungary) sweet corn hybrids] were sown on 29 April 2021. The temperature was ideal for the growth of the maize and the corn smut proliferation because it was between 10 °C and 25 °C during the experiment (Figure 1). 

A randomized complete block design was used as the experiment design. The size of the plots was 3 × 5 (vertical × horizontal) meters, and the soil disinfectant Bomber (tefluthrin) was sprayed into the rows in one pass with the sowing. A total of 4 corn rows/plots were sown with 25 plants per row. Plants were sown 5–6 cm deep, with 75 cm row spacing, and 20 cm seedling spacing. Plant irrigation was continuous, due to a drought watering took place every 2–3 days with 40–50 milliliters of water. Weed control was carried out by Principal Plus (nicosulfuron + dicamba + rimsulfuron) and insecticide treatment was carried out with Karate Zeon 5 CS (lambda-cyhalothrin). Fungicide was not used during the experiment.

The inoculum was created from the infected cobs using the method by Szőke and Tóth [61] under laboratory conditions at the laboratory of the Institute of Plant Protection, at the University of Debrecen. The infected corn cobs were collected from fields, and then the teliospores were sprayed on the corn smut-specific substrate. Then the pure culture was created. Next, the pathogen was replicated in the liquid-specific substrate and the dilution series (one -, ten -, one hundred -, one thousand -, ten thousand -, one hundred thousand-, and one million-fold) were made. Twenty-two strains were created and the first, seventh, and twelfth strains were compatible with each other. For the infection, the monosporidial strains were propagated on a liquid medium, grown for 48 h, and then mixed into a ratio of 1:1 before the inoculation. The cell numbers were set up to 2500, 5000, and 10,000 sporidia/cm^3^ in the Burker chamber. The infection was carried out at the vegetative stage (4–5 leaf stage, 3 June 2021) and at the generative stage (at the beginning of cob development, 29 June 2021). The biochemical and morphological parameters were measured at 7 and 14 days after the pathogen infection (DAPI)—because the symptoms appeared 7 days after the pathogen infection, the plants perished more at 14 days than after the pathogen infection—and the mineral contents and quality parameters were determined at 21 days after the pathogen infection (DAPI). The tumors surfaced on cobs at 21 days after the pathogen infection (DAPI).

### 2.2. Amount of Photosynthetic Pigments

The relative chlorophyll content was measured in the fourth leaf of sweet corn leaves with a SPAD-502+ Chlorophyll Meter (Minolta, Japan). The amount of the photosynthetic pigments was determined from the fourth leaves after the relative chlorophyll content measurement by the method of Moran and Porath [62], which was reformed by Wellburn [63]. Fifty mg of fresh leaf sample was collected from the fourth leaf and dissolved in 5 mL N, N-dimethylformamide at 4 °C for 72 h. The absorbance of the extract was measured spectrophotometrically and adjusted to wavelengths of 470, 647, and 664 nm. (Nicolet Evolution 300 UV-Vis Spectrometer; Thermo Fisher Scientific, Waltham, MA, USA). 

### 2.3. MDA Content Determination

The MDA concentration was determined fluorometrically from the fifth leaf [64]. The leaf sample (0.1 g) was pounded and homogenized with 1 mL 0.25% (*w*/*v*) thiobarbituric acid (TBA) and 10% (*w*/*v*) trichloroacetic acid (TCA). After the sample was centrifuged at 10,800× *g* for 25 min at 4 °C, 0.3 mL, the supernatant was transferred into the Eppendorf tube which contains 0.7 mL 5% (*w*/*v*) TBA and 20% (*w*/*v*) TCA. After the vortexing, the mixture was heated in a thermoshaker (Bioshan TS-100) at 95 °C for 30 min before being immediately cooled on ice. The absorbance was taken at 532 and 600 nm (Nicolet Evolution 300 UV-VIS Spectrometer) and the concentration of MDA was determined with the use of 155 mM^−1^ cm^−1^ as extinction coefficient.

### 2.4. Activities of Antioxidant Enzymes

The leaf samples were prepared using the method described by Pukacka and Ratahczak [65], and the activities of ascorbate peroxidase (APX) and guaiacol peroxidase (POX) were measured. Leaf samples (0.2 g) were frozen in liquid nitrogen and ground in a 1 mL 50 mM potassium phosphate buffer (pH 7.0) containing 2% (*w*/*v*) PVP, 1 mM ascorbate, 0.1% (*v*/*v*) Triton X-100, and 1 mM ethylenediaminetetraacetic acid (EDTA). The homogenates were centrifuged at 15,000× *g* for 20 min at 4 °C. The supernatant was collected and kept on ice until further processing. The activities of antioxidant enzymes were determined from the fifth leaves of the hybrids.

The APX activity was measured by the decrease in optical density due to ascorbic acid [66]. The final volume of the assay was 1 mL (550 µL of 50 mM potassium phosphate buffer (pH 7.0), 200 µL H_2_O_2_ (0.1 mM), 150 µL sodium ascorbate (0.5 mM), 50 µL EDTA (0.1 mM EDTA) and 50 µL sample extract). The ascorbate oxidation (decreased absorbance) was measured at 290 nm for 5 min at 20 °C compared to a blank that contained a phosphate buffer in place of the enzyme extract and the APX activity was calculated with the use of 2.8 mM^−1^ cm^−1^ as the extinction coefficient.

Zeislin and Ben-Zaken’s [67] method was used for the determination of POX activity. A mixture of 50 µL 0.2 M H_2_O_2_, 100 µL 50 mM guaiacol, 340 µL purified water, 490 µL 80 mM phosphate buffer (pH 5.5), and 20 µL enzyme extract was created. The produced concentration of tetraguaiacol was used to calculate POX activity. The reaction compound’s absorbance was measured at 470 nm for 3 min at 30 °C. In the blank, the sample extract was replaced with a 50 mM phosphate buffer. The extinction coefficient of 26.6 mM^−1^ cm^−1^ was utilized to estimate tetraguaiacol concentration.

The photochemical reduction of nitro blue tetrazolium chloride (NBT) was used to evaluate SOD activity [68]. The final volume of the assay was 4 mL containing 50 mM phosphate buffer (0.1 mM EDTA, 1% PVPP (*w*/*v*), 1 mM phenylmethylsulfonyl fluoride (PMSF) at pH 7.8). The homogenates were centrifuged at 10,000× *g* for 15 min. Twenty-five µL of plant extract, 25 µL of NBT (9 mM), 25 µL of riboflavin (0.25 mM), 250 L of methionine (0.16 M), and 2.675 mL of phosphate buffer (pH 7.8, 50 mM) were mixed and held at room temperature for 15 min. One SOD unit was defined as 50% inhibition of NBT. The absorbance was read at 560 nm. The blank contained 2.7 mL of phosphate buffer without plant extract.

### 2.5. Determination of Proline

Ninhydrin and acetic acid were used for proline concentration measurement [69]. Fresh wheat leaves (0.1 g) were homogenized with liquid nitrogen before being mixed with 2 mL 70% (*v*/*v*) ethanol. The 1 mL reaction mix (1% ninhydrin in 60% (*v*/*v*) acetic acid) was added to 500 µL of ethanolic extract, incubated at 95 °C for 20 min, cooled on ice, and centrifuged at 12,000× *g* for 1 min. The absorbance was read at 520 nm. Solutions of pure proline were used to calibrate the assays. The proline concentration was measured from the fifth leaves of the hybrids.

### 2.6. Measurement of Morphological Parameters

A sliding caliper was used to measure the diameter of the stem between the second and third nodes.

The plant’s height was measured from the peat surface to the tip of the youngest leaf.

The cob length was measured with a tape measure from the beginning of the cob to the end of the cob. The cob diameter was measured with a caliper before the corn shelling. After the shelling, 100 grains were counted, the kernel’s weight was weighed fresh and after drying, the dry weight was subtracted from the fresh weight. Drying took place at 65 °C for 3 days in an exsiccator. After the sampling, the cob mass was weighed on an analytical scale; in the case of infected plants, the infected cob mass was first weighed, then after the tumors were removed, the tumors were weighed, and the tumor’s weight was subtracted from the cob’s weight.

### 2.7. Measurement of the Quality Parameters of Grains

The quality parameters of the grains were determined by the method of Csapó et al. [70]. Nitrogen and protein content was determined by the method of Kjeldahl. The 5 g corn grain samples were digested at 420 °C in sulphuric acid (H_2_SO_4_) for 30 min. The end of digestion was indicated by the discoloration of the solution. Potassium sulfate (K_2_SO_4_) was then added, and ammonium sulfate (NH_4_) was formed. The volatile ammonia formed was removed from the solution by steam distillation, and the distillate was collected in 0.1 M hydrochloric acid (HCl). It was then titrated with 0.2 M NaOH until the color changed to red. 

The nitrogen and protein contents were calculated by these formulae:

(1) $$\mathrm{nitrogen}\%=\frac{\left(S\;-\;L\right)\times 0.0028016\times 100}{b}$$

S = amount of 0.1 M H_2_SO_4_ (mL) in the volumetric flask.
L = amount of 0.2 M NaOH (mL) consumed for the back titration of sulphuric acid.
B = Sample weight (g).
0.0028016 = nitrogen content which is corresponding to 1 mL of 0.1 M sulphuric acid quantity (g).

protein% = nitrogen% × 6.25(2)

6.25 = conversion factor.

To determine the ash content, 5 g of corn grain was measured and placed in the ashing crucible (which was measured before taking the plant sample) and placed in the drying oven (with a temperature of 550 °C) for 3 h. Then it was cooled in the desiccator and then weighed. The ash content was calculated with this formula:

(3) $$\mathrm{ash}\%=\frac{m1-m2}{m0}\;\times \;100$$

*m*_0_ = the plant sample weight (g).
*m*_1_ = the plant sample and ashing crucible weight (g).
*m*_2_ = the ashing crucible weight (g)

To determine the fiber content, 2 g cob grain was weighed, and 150 mL distilled water and 50 mL 0.51 M sulphuric acid were added and boiled for 30 min. Then 50 mL of cold distilled water was added, allowed to cool to room temperature, then filtered through a silk sieve. The residue was washed with warm water to make it an acid-free solution, then returned to the beaker. After 50 mL potassium hydroxide was added and it was boiled for 30 min and washed again with 50 mL of cold distilled water. The residue was filtered on filter paper (which was dried at 105 °C for one hour and the weight was measured before the filtering) (B). Then the filter paper with residue was washed with acetone and dried at 105 °C for between five and eight hours. Then it was cooled in a desiccator and the weight was measured (A). The A-B is the fiber that contains ash. Then the fiber ash was weighed, first weighed the heated porcelain dish (D) and placed the filter paper containing the fiber in it. Then, it was cremated in a muffle furnace at 550 °C for three hours. It was taken to the desiccator and the weight was measured (C). The fiber content is calculated by the following formula:

(4) $$\mathrm{fiber}\%=\frac{a-b}{m}\;\times \;100$$

*a*: the fiber which contains ash weight (g), A–B.
*b*: the ash weight (g), C–D.
*m*: the sample weight (g).

To calculate the dry matter of the kernels, 5 g grain was measured and put in the porcelain dish, which was taken into the drying oven set to 105 °C for 4 h, and weighed. After the plant samples were transferred into the porcelain dish and it was put into the drying oven set to 105 °C for 24 h and weighed. The dry matter content is calculated by the following formula:

dry matter% = A − B ∗ 100(5)

A: Dried porcelain dish weight (g) with plant samples.
B: Dried porcelain dish weight (g) without plant samples.

To determine crude fat content, 5 g of grain was measured in a fat-free extraction tube and the tube was sealed with fat-free absorbent cotton. The tube containing the sample to be analyzed was placed in the middle part of the extraction apparatus and then connected to the flask that contained 4–5 pieces coarse gravel, pre-weighed (m^2^), and filled with n-hexane or petroleum ether filled to 3/4 full. After installing the cooler, the samples were extracted for 6 h by heating them so that the sample would only contact fresh solvent (at least ten times per hour). After six hours of extraction, the tube with the sample was removed and the solvent in the flask was distilled into the middle part while being continuously removed from there. The flasks with the fat and solvent residues were placed in the drying oven at a temperature of 98 °C for one hour, then cooled in an exsiccator and weighed. The raw fat content is calculated by the following formula:

(6) $$\mathrm{crude}\;\mathrm{fat}\%=\frac{m1-m2}{m0}\;\times \;100$$

*m*_0_: the sample weight (g).
*m*_1_: the flask, pumice stone, and dried sample weight (g).
*m*_2_: the flask and pumice stone weight (g).

### 2.8. Measurement of Mineral Elements Concentration

The collected corn kernels were dried at 30 °C for four days, then 1 g sample was measured and ground. Fifteen ml HNO_3_ (65% *v*/*v*) was added to 1 g of the sample and incubated overnight at room temperature. The materials were then pre-digested for 30 min at 60 °C. Finally, 5 mL H_2_O_2_ (30 m/m%) was added for 270 min while the solution was boiled at 120 °C. The solutions were prepared to a volume of 50 mL, homogenized, and filtered using Filtrak 388 filter paper. Analytical grade HNO_3_ (65%) solution (manufactured by Merck) was utilized for solution preparation. Merck solutions, as well as analytically pure compounds, were used in the manufacturing of basic solutions.

An OPTIMA 3300 DV type ICP-OES was used for the analytical definition [71].

### 2.9. Method of Statistical Analysis

The IBM SPSS Statistics 25 software (Armonk, NY, USA) was used for statistical analysis. The normality of data was determined by Kolmogorov—Smirnov and Shapiro—Wilk tests [72]. One-way and three-way ANOVA [73] were used for the analysis, and the means were compared by the Tukey-HSD test [74] at a significance level of 5% (*p* ≤ 0.05) because of the normal distribution of data. The number of replicates was five per treatment per investigated parameter. The statistical difference was labeled by small characters (a, b, c, d, e, and f) in the text. For cluster analysis, the samples were standardized with the descriptive group. Thus, we obtained the Z values needed for the cluster analysis. Then the K-means cluster analysis was performed based on the treatments with the Z values and one-way ANOVA was carried out for the evaluation of the significant differences among the created clusters. The Pearson correlation also was performed for linear correlation that measures the strength and direction of the relationship between two variables. 

## 3. Results

Three-way ANOVA was performed to evaluate the significance of the independent variables [hybrid (H), treatment (T) as inoculum concentration, and sampling time (S)] separately and their interactions. The hybrid impact was significant for Chl-b, MDA, APX, POX, plant height, and stem diameter. Treatment impacts were significant for measured characteristics except for chlorophyll-b and carotenoids. Sampling time also had substantial effects on all physiological parameters (SPAD, MDA, APX, POX, SOD, proline, plant height, and stem diameter), except Chl-*a*, -*b*, and carotenoids. There were significant interactions between the hybrid and the treatment for Car, MDA, APX, SOD, and plant height. The interaction between hybrid and sampling time was significant for Chl-*a*, MDA, POX, plant height, and stem diameter. Significant interactions were observed between treatment and sampling time for MDA, APX, POX, SOD, proline, and plant height. The three-way interaction (hybrid x treatment x sampling time) only had a significant impact on Chl-*a*, -*b*, Car, APX, proline, plant height, and stem diameter (Table 1).

**Figure 2:**
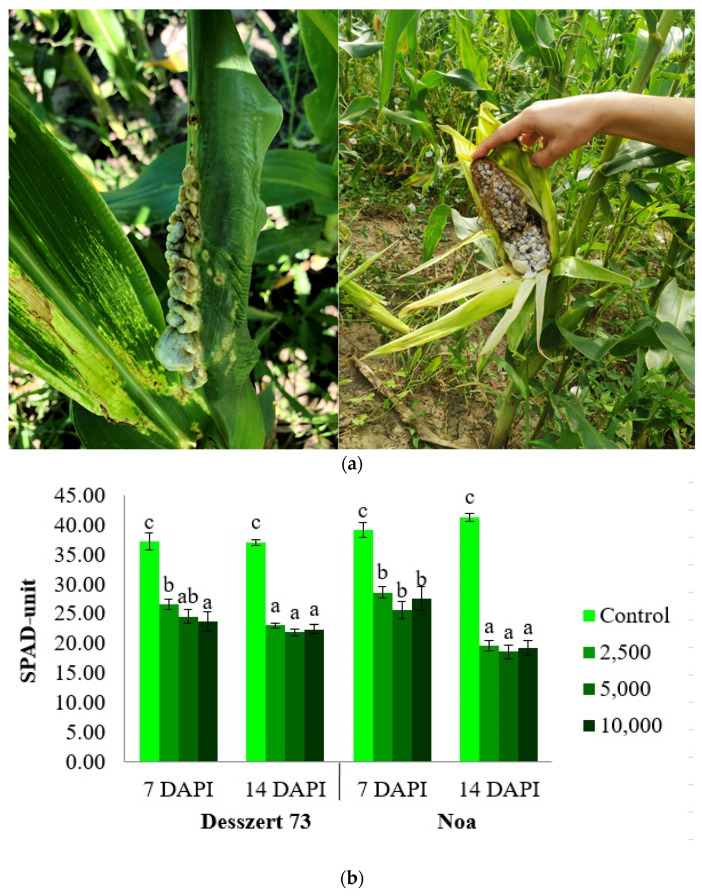
(**a**) Corn smut infected leaves and cob at 10,000 sporidia/mL on Desszert 73 hybrid. (**b**) The relative chlorophyll content (SPAD-unit) of the four leaves of corn smut infected Desszert 73 and Noa hybrids at 7 and 14 DAPI (mean ± SD, n = 25). The data were evaluated by one-way ANOVA followed by the Tukey-HSD test at 0.05 to determine significant differences indicated by different letters (a, b, and c). DAPI: days after the pathogen infection.

The applied fungal treatments (2500, 5000, and 10,000 sporidia/mL) significantly reduced the SPAD-unit (*p* < 0.05) (40%, 52%, and 57%, respectively) in the Desszert 73 and Noa hybrid (37, 53, and 42%, respectively) at 7 DAPI. Similarly, these fungal treatments substantially (*p* < 0.05) reduced relative chlorophyll contents (61%, 69%, and 65% in Desszert 73; and 110%, 122%, and 113% in Noa hybrid) at 14 DAPI (Figure 2).

**Figure 3:**
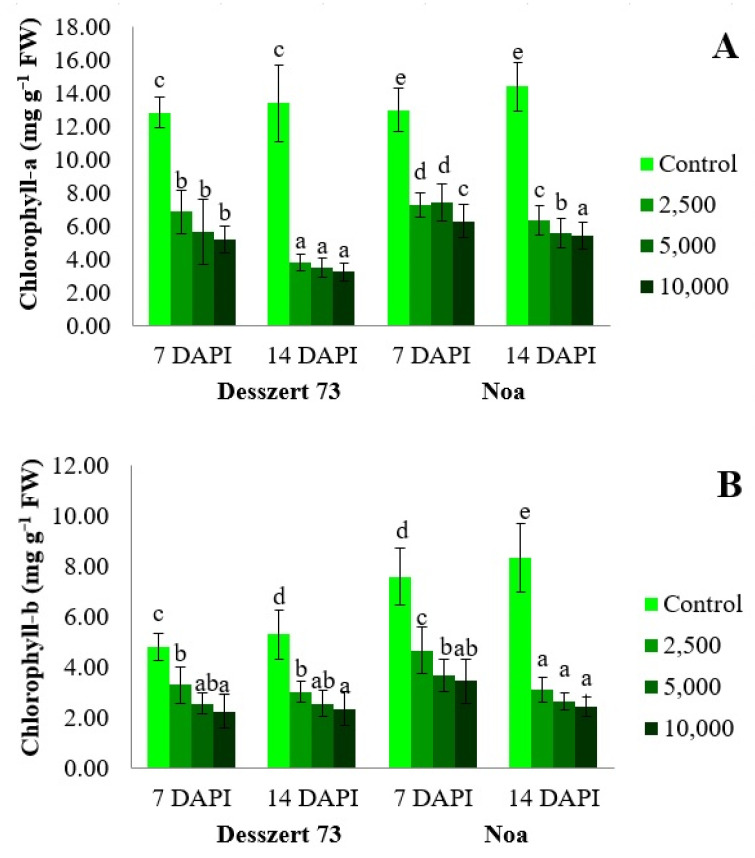

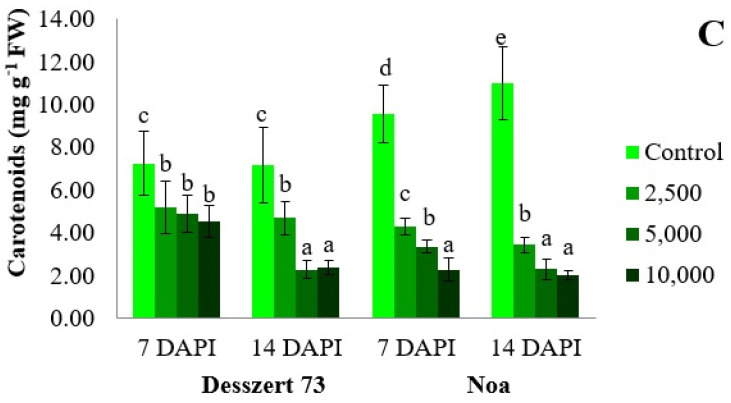
(**A**) The effects of corn smut infection on chlorophyll-*a* (mg g^−1^ FW) (mean ± SD, n = 5), (**B**) The effects of corn smut infection on chlorophyll-*b* (mg g^−1^ FW) (mean ± SD, n = 5), (**C**) The effects of corn smut infection on carotenoids (mg g^−1^ FW) (mean ± SD, n = 5) of the fourth leaves of Desszert 73 and Noa hybrids at 7 and 14 DAPI. The data were evaluated by one-way ANOVA followed by the Tukey-HSD test at 0.05 to determine significant differences indicated by different letters (a, b, c, d, and e). DAPI: days after the pathogen infection, FW: fresh weight.

Since the relative chlorophyll content is an index, the amounts of photosynthetic pigments (chlorophyll-*a*, chlorophyll-*b*, and carotenoids) were also measured. The increased spore concentration (2500, 5000, and 10,000 sporidia/mL) had significant effects on chlorophyll-*a* in both hybrids at both sampling times. Chlorophyll-*a* content of Desszert 73 and Noa significantly decreased (*p* < 0.05) when infected by the three corn smut concentrations (87%, 127%, and 146% in Desszert 73, and 79%, 75%, and 106% in Noa) at 7 DAPI compared to the control plants. At the second sampling time (14 DAPI), the reductions were 254%, 286%, and 316% for Desszert 73 and 127%, 160%, and 167% for Noa, respectively. Noa had the most significant reduction when the highest sporidium treatment was applied (means for at 7 and 14 DAPI at 10,000 sporidia/mL followed by different letters) (Figure 3A). 

Corn smut infection also had negative effects on the amounts of chlorophyll-*b* in the Desszert 73 and Noa hybrids (*p* < 0.05). Increased concentration of sporidia diminished chlorophyll-*b* by 46%, 89%, and 116% in Desszert 73 and 63%, 107%, and 121% in Noa compared to the control plants at 7 DAPI. Furthermore, the reduction was 76%, 108%, and 127% in Desszert 73 and 170%, 214%, and 244% in Noa hybrids at 14 DAPI (Figure 3B).

The carotenoid content was reduced at 7 DAPI by 40% (*p* = 0.016) and 123% (*p* = 0.006) in the Desszert 73 and Noa hybrids treated 5000 sporidia/mL; 49% (*p* = 0.023) and 185% (*p* = 0.006) in Desszert 73 and Noa hybrids treated with 10,000 sporidia/mL. Similarly, at 14 DAPI, carotenoid content was also significantly reduced under 2500, 5000, and 10,000 sporidia/mL in Desszert 73 [53% (*p* = 0.013), 222% (*p* = 0.005), and 216% (*p*= 0.004)] and Noa [380% (*p* = 0.000), 203% (*p* = 0.030), and 450% (*p* = 0.000] (Figure 3C).

**Figure 4:**
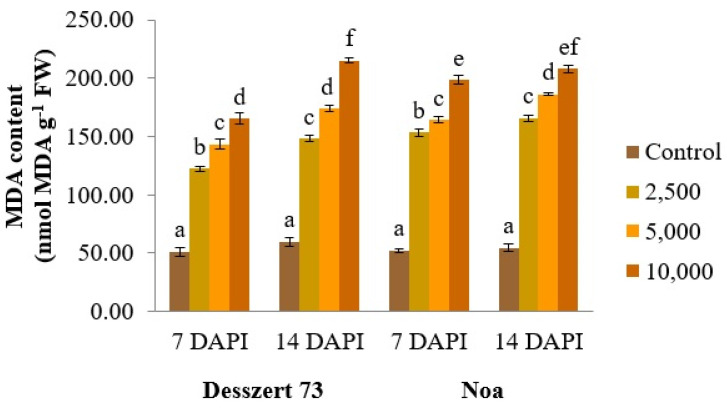
The MDA content (nmol MDA g^−1^ FW) of the fifth leaves of corn smut infected Desszert 73 and Noa hybrids at 7 and 14 DAPI (mean ± SD, n = 5). The data were evaluated by one-way ANOVA followed by the Tukey-HSD test at 0.05 to determine significant differences indicated by different letters (a, b, c, d, e, and f). DAPI: days after the pathogen infection. FW: fresh weight, MDA: malondialdehyde.

The respective corn smut treatments significantly increased (*p* ≤ 0.001) the MDA content of Desszert 73 and Noa hybrids’ leaves (140%, 181%, 224% and 194%, 215%, and 280% reduction) at 7 DAPI. The MDA content was significantly (*p* ≤ 0.001) increased at 14 DAPI in Desszert 73 and Noa infected with the three concentrations of corn smut (147%, 191%, and 260% increase; 205%, 242%, and 282% increase) in the infected plants due to the different concentration of the inoculum at 14 DAPI (Figure 4).

**Figure 5:**
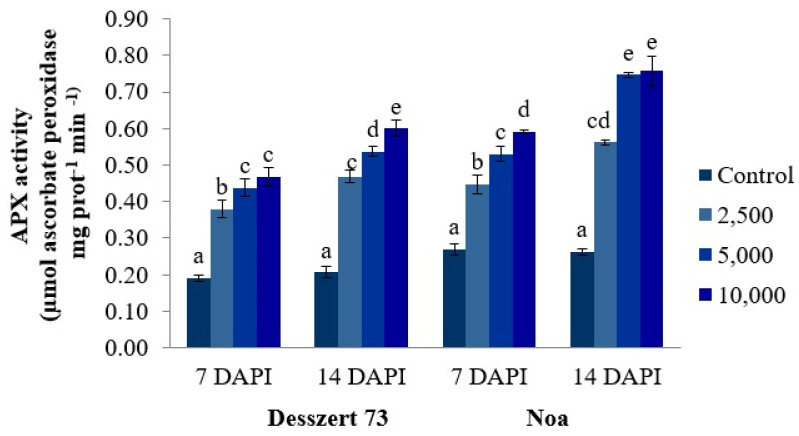
The effect of corn smut infection on the APX activity (µmol ascorbate peroxidase min^−1^ mg prot^−1^) of the fifth leaves of Desszert 73 and Noa hybrids at 7 and 14 DAPI (mean ± SD, n = 5). The data were evaluated by one-way ANOVA followed by the Tukey-HSD test at 0.05 to determine significant differences indicated by different letters (a, b, c, d, and e). DAPI: days after the pathogen infection.

The APX activity in the leaves of Desszert 73 and Noa hybrids significantly (*p* ≤ 0.001) increased with infection intensity (100%, 132%, 147%, and 67%, 96%, and 119%, respectively, significant differences under 2500, 5000, and 10,000 sporidia/mL) for Desszert and Noa hybrids at 7 DAPI. Similarly, all applied treatments increased APX activity in Desszert 73 and Noa hybrids (124%, 157%, 186%, 115%, 188%, and 192%, respectively) compared to the control plants (*p* ≤ 0.000) at 14 DAPI (Figure 5).

**Figure 6:**
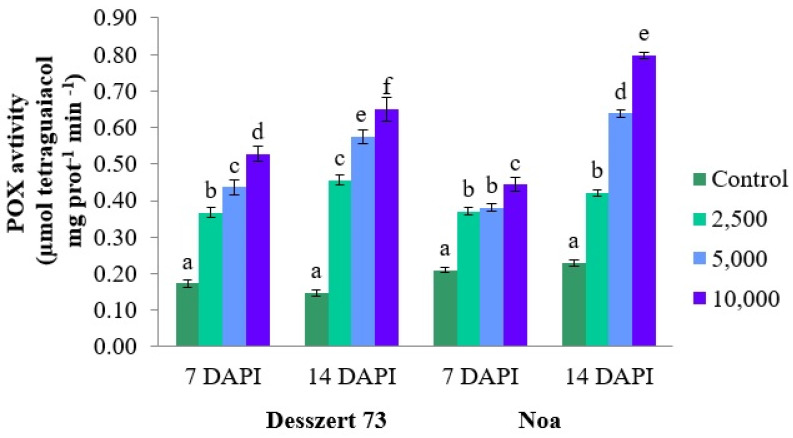
The effect of corn smut infection on the POX activity (µmol tetraguaiacol min^−1^ mg prot^−1^) of the fifth leaves of Desszert 73 and Noa hybrids at 7 and 14 DAPI (mean ± SD, n = 5). The data were evaluated by one-way ANOVA followed by the Tukey-HSD test at 0.05 to determine significant differences indicated by different letters (a, b, c, d, e, and f). DAPI: days after the pathogen infection.

**Figure 7:**
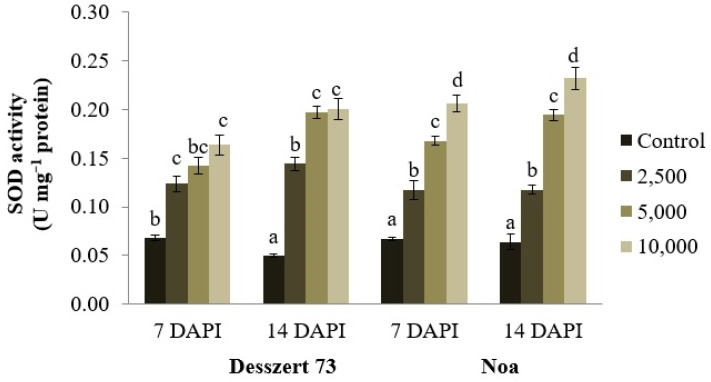
The effect of corn smut infection on the SOD activity (U mg^−1^ protein) of the fifth leaves of Desszert 73 and Noa hybrids at 7 and 14 DAPI (mean ± SD, n = 5). The data were evaluated by one-way ANOVA followed by the Tukey-HSD test at 0.05 to determine significant differences indicated by different letters (a, b, c, and d). DAPI: days after the pathogen infection.

The POX activity in the leaves of the infected two hybrids was also elevated due to the difference in sporidia of corn smut at both sampling dates. The POX activity increased substantially (*p* ≤ 0.001), at 7 DAPI, for corn smut infected (2500, 5000, and 10,000 sporidia/mL) Desszert 73 (118%, 159%, and 212%) and Noa (76%, 81%, and 110%). At the second sampling (14 DAPI), POX increased by 207%, 287%, and 333% (*p* ≤ 0.001) in Desszert 73 and 83%, 178%, and 243% (*p* ≤ 0.001) in the Noa hybrid (Figure 6). The increased concentration of corn smut (2500, 5000, and 10,000) induced higher SOD activity in Desszert 73 (1.82; 2.09 and 2.40 times higher; *p* ≤ 0.001) and Noa (1.75; 2.51 and 3.08 times higher; *p* ≤ 0.001) hybrids at 7 DAPI. At 14 DAPI, this activity was still significantly (*p* ≤ 0.001) high 2.89; 3.84, and 4.02 times higher in Desszert 73; 1.49; 3.05, and 3.63 times higher in Noa (Figure 7).

**Figure 8:**
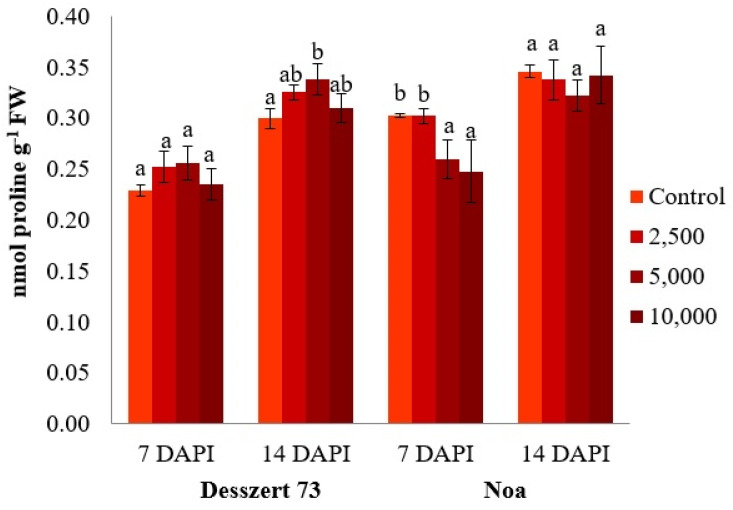
The proline concentration (nmol proline g^−1^ FW) of the fifth leaves of corn smut infected Desszert 73 and Noa hybrids at 7 and 14 DAPI (mean ± SD, n = 5). The data were evaluated by one-way ANOVA followed by the Tukey-HSD test at 0.05 to determine significant differences indicated by different letters (a, and b). DAPI: days after the pathogen infection, FW: fresh weight.

Corn smut infection did not have a constant effect on the proline concentration in the leaves of sweet corn hybrids. At the first sampling time (7 DAPI), there were no significant differences in proline across different sporidia at Desszert 73. This, however, increased significantly at 5000 sporidia/mL at 14 DAPI in Desszert 73. Proline was significantly lower in Noa infected with 5000 and 10,000 sporidia/mL (15% and 18%, *p* ≤ 0.050). At 14 DAPI, proline content was not significantly affected by the different sporidia in Noa (Figure 8).

**Figure 9:**
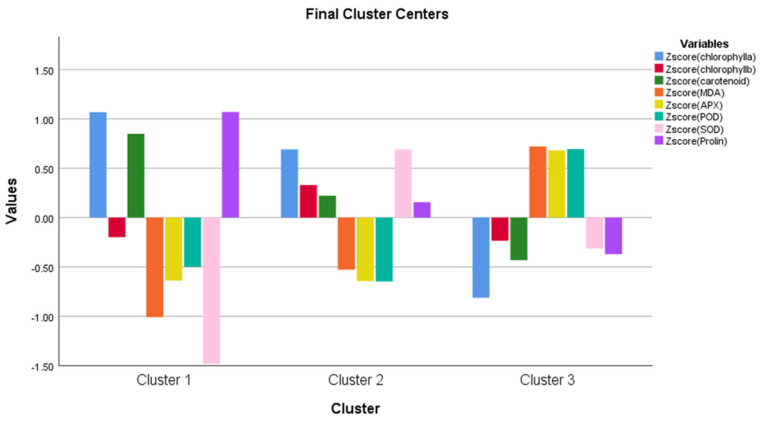
K-mean cluster analysis for chlorophylls (chlorophyll-*a*, and *b*), carotenoids, malondialdehyde (MDA), ascorbate peroxidase (APX), guaiacol peroxidase (POD), superoxide dismutase (SOD), and proline.

The K-mean cluster analysis created three groups (cluster 1, cluster 2, and cluster 3). It compares the clusters (groups) to an average. Where the values are positive the cluster is above the average, where the values are negative the cluster is below the average. For chlorophyll-*a*, the first and second groups were above average, and the third group was below average. For chlorophyll-*b*, groups one and three are below average, and group two is above average. For carotenoids, the first group (cluster) is above average, the second and third are below average. For MDA, APX, and POD, the first and second clusters are below average, and the third is above average. At SOD, the first and third groups are below average, and the second is above average. For proline, the first group is above average, and the second and third are below average (Figure 9).

The one-way ANOVA analysis shows that there is a significant difference among the clusters presented in Figure 9 (Table 2).

Chlorophyll-*a* had a significant positive correlation with carotenoids and proline, and a negative correlation with MDA, APX, and POX. Chlorophyll-*b* was negatively correlated with APX and POX. Carotenoids were negatively correlated with MDA. MDA was negatively correlated with SOD and proline and strongly positively correlated with APX and POX. There was a strong positive correlation between APX and POX. In addition, there was a significant negative correlation between POX and SOD (Table 3).

The effects of the corn smut infection on the host plants’ morphological parameters (plant height and stem diameter) were also investigated. Plant height was reduced in both hybrids by all treatments for corn smut infection. Desszert 73 plant height was reduced by 21%, 31%, and 24%, and that of Noa was reduced by 21%, 23%, and 29% due to the 2500, 5000, and 10,000 sporidia number of corn smut infection at 7 DAPI. Similarly, in the second sampling (14 DAPI), the corn smut treatments (in their increasing order) decreased plant height of Desszert 73 (45%, 60%, and 67%) and Noa (23%, 37%, and 38%) (Table 4).

As a result of the increasing corn smut infection (2500, 5000, and 10,000 sporidia/mL), both hybrids had increased stem diameter (15%, 24%, and 23% increase for Desszert 73; 10%, 11%, and 17%, increase for Noa). Stem diameter for Desszert 73 and Noa also increased significantly with sporidium treatment (control means represented by different letters) (Table 3).

**Figure 10:**
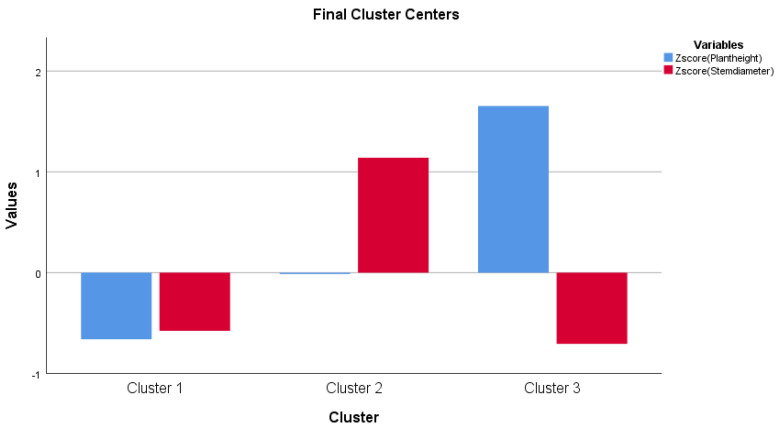
K-mean cluster analysis for plant height and stem diameter.

Figure 10 shows that the plant height’s first and second clusters are below the average, while the third is above average. Regarding the stem diameter, the first and third clusters are below the average, and the second is above the average. Based on the one-way ANOVA analysis, there is a significant difference among the three clusters (data are not shown). Furthermore, there is no significant correlation between plant height and stem diameter according to the Pearson correlation (results are not shown). 

The negative effects of corn smut infection were also observed at the generative stage. Only the 10,000 sporidia/mL of corn smut caused symptoms (tumor growth) on the cobs of both hybrids at 21 DAPI. This infection significantly reduced the cob length in Desszert 73 and Noa (19% and 30%, *p* ≤ 0.050) at 21 DAPI. The cob diameter was not affected by the corn smut infection. The 10,000 sporidia/mL infected plants had significantly lower kernel weights (41% reduction in Desszert 73, and 18% reduction in Noa hybrid; *p* ≤ 0.050) at 21 DAPI. The 100 grains fresh weight was significantly reduced in the Desszert 73 and Noa hybrids’ infected plants (by 21% and 17% compared to the control plants; *p* ≤ 0.050) by the corn smut infection (10,000 sporidia/mL) at 21 DAPI. The 100 grains’ dry weight was also reduced by 51% and 59% (*p* ≤ 0.050) in both hybrids (Table 5). 

**Figure 11:**
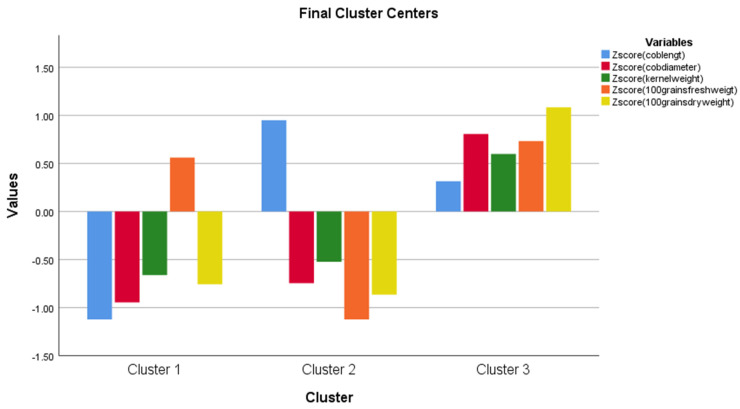
K-mean cluster analysis for cob length, cob diameter, kernel weight, 100 grains fresh and dry weight.

The cluster analysis shows that the 100 grains’ fresh weight is above the average, while every other kernel parameters are below the average in the first cluster. While the cob length is above the average in the second, and all of the kernel parameters are above the average in the third cluster (Figure 11). In addition, the one-way ANOVA analysis shows that there is a significant difference among the clusters presented in Figure 11 (Table 6).

**Figure 12:**
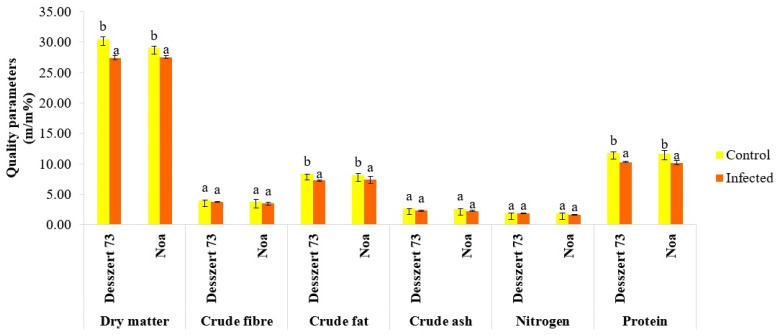
The effects of corn smut infection on the quality parameters of the kernel (*m/m*%) of Desszert 73 and Noa hybrids at 21 DAPI (mean ± SD, n = 5). The data were evaluated by one-way ANOVA followed by the Tukey-HSD test at 0.05 to determine significant differences indicated by different letters (a, and b). DAPI: days after the pathogen infection.

The Desszert 73 and Noa corn smut-infected kernel had lower dry matter (10% and 5%), crude fat (9% and 11%), and protein (13% and 15%) (*p* ≤ 0.050) contents compared to the control, non-infected. The infection did not affect crude fiber, crude ash, and nitrogen contents (Figure 12). 

**Figure 13:**
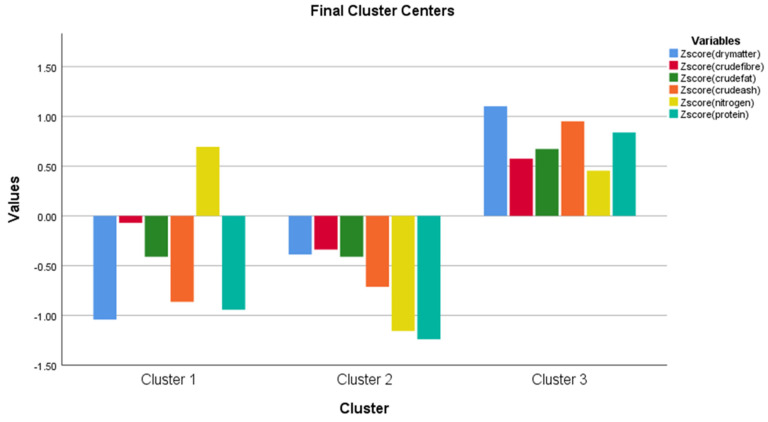
K-mean cluster analysis for the quality parameters of kernel (dry matter, crude fiber, crude fat, crude ash, nitrogen, and protein (m/m%) of Desszert 73 and Noa hybrids at 21 DAPI.

The K-mean cluster analysis created three groups (cluster 1, cluster 2, and cluster 3). The dry matter, crude fiber, crude fat, and protein are below the average at the first cluster, while these are above the average at the third cluster. For nitrogen, the first and third clusters are above the average, and the second is below the average (Figure 13). There are significant differences among the clusters based on one-way ANOVA (results are not shown).

Infection reduced the Mg and Mn content in both hybrids kernel compared to the control (15% and 25% in Desszert 73 and 8% and 15% in Noa hybrid, respectively; *p* ≤ 0.050). The concentration of Al, Ca, and S were significantly increased by 182% (*p* ≤ 0.000), 12% (*p* = 0.038), and 7% (*p* = 0.045) in Desszert 73 and by 105% (*p* = 0.002), 8% (*p* = 0.045), and 14% (*p* = 0.041), in the Noa hybrid, respectively, due to the corn smut infection. The infected kernel of Desszert 73 had lower level of B, P, and Zn by 11%, 12%, and 6% (*p* ≤ 0.050), respectively, compared to the control, non-infected. Corn smut infection significantly decreased K concentration (5% reduction; *p* ≤ 0.050) but increased the Na concentration (13% increase; *p* ≤ 0.050) in the Noa hybrid’s kernel (Table 7).

**Figure 14:**
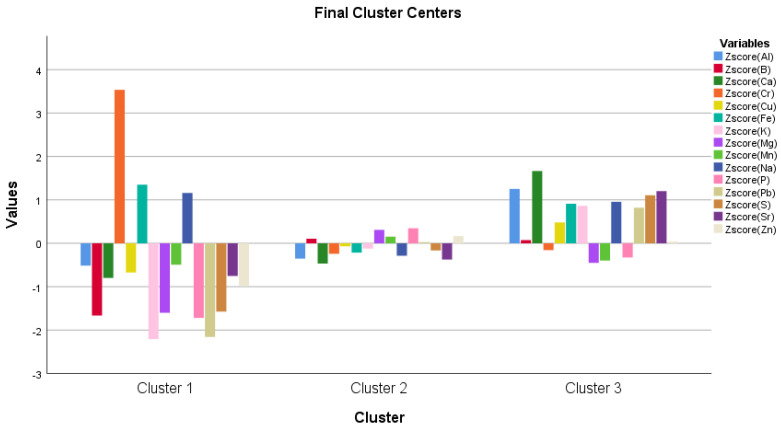
K-mean cluster analysis for on the kernel’s elements concentration (Al, B, Ca, Cr, Cu, Fe, K, Mg, Mn, P, Pb, S, Sr, and Zn) of Desszert 73 and Noa hybrids at 21 DAPI.

In addition, K-mean cluster analysis shows that for Al, Ca, Cu, K, S, and Sr, the first and second clusters are below the average, while the third is above the average. For B, Mg, Mn, P, Pb, and Zn, the first cluster is below the average, the second and third are above the average. For Cr and Fe, the first and third clusters are below the average, and the second is above the average (Figure 14). Based on one-way ANOVA analysis, there are significant differences among the clusters for Al, Ca, Cr, K, Na, Pb, S, and Sr (results are not shown).

## 4. Discussion

The effects of different corn smut inoculum treatments (2500, 5000, and 10,000 sporidia/mL) on the biochemical (amounts of photosynthetic pigments, MDA content and proline concentration, activities of APX, POX, and SOD enzymes), morphological (plant height and stem diameter), mineral contents, and quality parameters were determined. The first goal of this study was to examine the tumor formation at the V4–V5 phenological stage because the first hypothesis was that there is no tumor formation at low (2500 sporidia/mL) inoculation. Based on the outcome of the inoculation, tumor formation occurred at lower (2500 and 5000 sporidia/mL) and at high (10,000 sporidia/mL) corn smut sporidia numbers, too. The second hypothesis of this research was that the corn smut infection adversely affects the morphological, physiological, biochemical, and quality parameters, as well as the element content and other quality characteristics of grains irrespective of the lower dosage. The third hypothesis was that the 10,000 sporidia/mL has more negative impacts on the measured parameters relative to the 2500 and 5000 sporidia/mL treatments. The further goal was to examine which phenological stage (V4–5 or V7) was more susceptible to corn smut infection. 

The first visible symptom of corn smut infection is the yellowing of the leaves which can be measured with the relative (SPAD Units) and absolute photosynthetic content (mg g^−1^). The relative chlorophyll content (Figure 2) and the amount of photosynthetic pigments (Figure 3) of the infected plants were reduced in the fourth leaves in both hybrids at 7 and 14 DAPI. However, there were no significant differences among the treatments in the relative chlorophyll content for both hybrids, at 14 DAPI. These results do not confirm our second hypothesis because the 10,000 sporidia/mL treatment had no significant effects relative to 2500 and 5000 sporidia/mL treatments (Table 1). This effect was also observed in other studies. Frommer et al. [75] measured lower SPAD- units in sweet corn hybrids infected with corn smut. Szőke et al. [76] found that the 5000 and 10,000 sporidia/mL of corn smut reduced the relative chlorophyll content of the fodder corn hybrid. The reduction of photosynthetic pigments due to corn smut infection was also found in previous studies [77,78]. The corn smut disease causes chlorosis and necrosis on leaves, and this is the reason why the chlorophyll content was reduced [79].

Malondialdehyde content is an indicator of the presence of oxidative stress. Various biotic stress factors increase the MDA content in plants [80]. Meena et al. [81] found high MDA content in the *Alternaria alternata*-infected tomato plants. The *Plasmodiophora brassicae*-infected pakchoi plants had also high MDA content [82]. Chen et al. [83] stated that the *Puccinia striiformis* f. sp. *tritici* infection raised the concentration of MDA in the wheat leaves. In this study, the different treatments for corn smut infection also increased the concentration of MDA in the fifth leaves drastically in both hybrids (Figure 4). This effect proves that the corn smut infection was successful because of the increased MDA concentration as an indication of stress conditions. Secondly, this data confirms the second hypothesis that the corn smut infection had significant impacts on the physiological parameters of corn. The MDA content increased with the increasing corn smut sporidia. The highest MDA content was observed in both hybrids at both sampling times under 10,000 sporidia/mL inoculum. This result confirms the third hypothesis because there were significances among the 10,000 and 25,000 and 5000 sporidia/mL treatments. In addition, the MDA content was significantly influenced by the hybrids, treatment, sampling times, and the interactions of any two factors. The interactions of hybrid × treatment × sampling time did not have any significant impact on this parameter (Table 1). The hybrids did not tolerate the corn smut infection at the vegetative stage, and this was not dependent on the concentration of corn smut. The reason for this is that sweet corn hybrids are very susceptible to corn smut infection and the lower concentration infection can also cause severe damage to sweet corn. 

The relationship between antioxidant enzyme activities and corn smut infection was also studied. Infection significantly increased the APX, POX, and SOD activities (Figure 5, Figure 6 and Figure 7) for both hybrids’ fifth leaves. Consistently with the second hypothesis, the activities of APX and POX were significantly affected by the hybrid (Noa had higher activities), treatment (5000 and 10,000 sporidia/mL had higher activities relative to 2500), sampling time (14 DAPI had higher values), and the interaction of treatment and sampling time. The SOD activity was influenced by treatment, sampling time, and the interaction of hybrid and treatment, and treatment and sampling time (Table 1). However, there is no convincing evidence that the higher concentration of 10,000 sporidia/mL has more adverse impacts on antioxidant enzyme activities compared to the lower (2500 and 5000 sporidia/mL) sporidia numbers treatment. The activity of POX was significantly higher at all treatments at both hybrids and both sampling times at 10,000 sporidia/cm^3^ treatments compared to the 2500 and 5000 sporidia/mL treatments. The APX activity was not significantly higher at 7 DAPI at Desszert 73 and at 14 DAPI at Noa when 10,000 sporidia/mL treatment was compared to the other two treatments. Significantly higher SOD activity was measured at Noa at both sampling times but not at Desszert 73. Similar studies also reported the effects of infection with various diseases on antioxidant enzyme activities. The Cucumber Green Mottle Mosaic Virus infection induced higher APX, POX, and SOD activities in cucumber plants [84]. The results of Kovács et al. [85] showed higher APX, POX, and SOD activities in European chestnut leaves infected with *Cryphonectria parasitica*. Barley plants infected with *Blumeria graminis* f. sp. *hordei* had higher APX and SOD activities compared with uninfected control plants [86]. The different pathogen infections produce different ROS and cause an oxidative burst [87]. Along with these progressions, plant antioxidant enzymes are activated after infection [88].

Proline content was also significantly elevated in the fifth leaf of Desszert 73 infected with 5000 sporidia/mL, at 14 DAPI. However, in Noa’s fifth leaf, the proline content was significantly lower in the infected plants with 5000 and 10,000 sporidia/mL at 7 DAPI as compared to control, non-infected plants (Figure 8). Furthermore, proline concentration was significantly affected by treatment, sampling time, the interaction of treatment and sampling time, and hybrid × treatment × sampling time (Table 1). Based on the proline data, there are no significant differences between 10,000, 2500, and 5000 sporidia/mL treatments. This means corn smut sporidia number did not change significantly the proline content of the host plant. A biotic factor may initially lower the proline content in the plants [89]. In this study, the proline content in the infected plants was high under field conditions. Proline content is affected by other factors such as UV radiation [90], temperature [91], heavy metals [92], and so on. Furthermore, since the experiment was conducted under field conditions, these factors could not be controlled. 

The applied treatments of corn smut infection also affected the plant height and stem diameter (Table 4) for both hybrids at both sampling times. The effect of the pathogen infection on the host plant height and stem diameter was also found in other studies. According to Szőke et al. [52], the corn smut infection decreased plant height and increased the stem diameter under greenhouse conditions at 7 DAPI. The tobacco mosaic virus decreased the plant height and stem diameter of pepper plants [93].

Adverse effects of the corn smut infection on the generative stage were also observed. Interestingly, only the 10,000 sporidia/mL caused symptoms on the infected cobs in both hybrids. Thus, the maize plants tolerated the lower concentrations (2500 and 5000 sporidia/mL) of the corn smut infection. This proves that the examined sweet corn hybrids are more susceptible to corn smut infection during the V4–5 stage than at the V7 stage. This is the first observation of these two (Desszert 73, and Noa) sweet corn hybrids’ sensitivity comparison based on the phenological stage. The corn smut infection significantly diminished the dry matter, fat, and protein contents of the kernel, cob length and diameter, and kernel-100 grains of fresh and dry weights in both hybrids (Figure 12 and Table 5). These results are similar to some previous studies’ results. According to Keszthelyi et al. [94], the corn smut infection decreased the protein, but not the fat content of a fodder corn hybrid’s kernel. They stated that kernel and grain weights were also diminished by the corn smut infection. They also showed that the dry matter, protein, fat, ash, and nitrogen contents were decreased in other fodder corn hybrids by the corn smut infection [55]. 

Contrary to the second hypothesis of the study, the impacts of corn smut infection were different considering different elements. Magnesium and Mn had lower concentration while Al, Ca, and S had higher concentration in both hybrids’ kernel at 21 DAPI. Boron, P, and Zn were reduced in Desszert 73, and K was reduced in the Noa hybrid’s kernel (Table 7). Thus, the successful effect of the infection may depend on the cultivar. These differences proved that there is an effect of a hybrid on the case of grains’ element contents as well. There are very few studies on the effects of plant pathogens on host plant element content. Shattuck et al. [95] showed that the Turnip mosaic virus did not alter mineral contents (Mg, Mn, Ca, Zn, B, and P) in rutabaga roots. Cesco et al. [96] reported that the *Plasmopara viticola*-infected plants had higher levels of the elements Ca, Fe, Mn, and Cu than control plants. Minerals can also influence the interaction between the pathogen and the host plant. The different mineral elements (K, Ca, Mn, Mg, and Zn) can increase the resistance of the host plant to different diseases [97].

The K-mean cluster analysis created three groups (cluster 1, cluster 2, and cluster 3) for measured parameters. The three clusters mean how different the three groups were from the average. The difference between the clusters was shown in the ANOVA table for a given parameter. The three groups were created based on the degree of freedom (df) in the cluster section for the created Z values, which is a recommendation of how many cluster groups should be created. This adds +/− 1. Of course, we would have liked to create more cluster groups, we created them, however, the created a higher number of groups and did not show a significant difference among the clusters for any of the parameters, so the cluster analysis makes no sense. That is why we stayed at the recommended 2 +/− 1 for the Z value, i.e., the three groups (Figure 9, Figure 10, Figure 11, Figure 13 and Figure 14).

## 5. Conclusions

The different concentrations of the corn smut infection had impacts on the physiological, morphological, quantity, and quality parameters of maize. In the vegetative stage, the hybrids did not tolerate the corn smut infection. Sweet corn is very susceptible to corn smut. Corn smut can cause severe damage regardless of the concentration of the pathogen. So, prevention is very important against corn smut. However, in the generative stage, only the concentration of 10,000 sporidia/mL of corn smut affected the content of corn cob elements and caused symptoms (tumor growth and galls). Thus, plants can better tolerate a lower concentration of infection at the generative stage. This proves that the examined sweet corn hybrids are more susceptible to corn smut infection during the V4–5 stage than at the V7 stage. This is the first observation of these two (Dessert 73, and Noa) sweet corn hybrids’ sensitivity comparison based on the phenological stage. The results partly prove our hypothesis. Relative chlorophyll content (SPAD unit) and photosynthetic pigments (chlorophyll-*a*, chlorophyll-*b*, and carotenoids) content did not change significantly when 10,000 sporidia/mL were used compared to 25,000 and 5000 sporidia/mL. In addition, the sampling time also did not have any significant effect on these characteristics. Furthermore, MDA content and POX activity of the leaves were significantly higher at 10,000 sporidia/mL relative to 25,000 and 5000 sporidia/mL. This proves that photosynthesis and related parameters are less affected during corn smut infection than the defense system of the host plants. Only 10,000 sporidia/mL infected the corn cob during the generative stage, so we do not make any conclusion related to the impacts of sporidia concentration.

With appropriate indirect protection, i.e., by avoiding mechanical damage, using insecticide treatments, and proper care of the crop area, we can reduce the amount of pathogen inoculum (infectious material) concentration, because, with appropriate indirect protection, we reduce the optimal environmental conditions for the pathogen, as well as the optimal conditions for the development of the cultivated plant we create optimal conditions for the development of the cultivated plants. The experiment proved that resistant and less susceptible hybrids can tolerate lower concentrations of goiter infection (2500 and 5000 sporidia/mL) without pathogenic symptoms.

## Figures and Tables

**Table 1:** Combined analysis of variance for measured parameters of two sweet corn cultivars with three treatments over two sampling times.

	Hybrid (H)	Treatment (T)	Sampling Time (S)	HxT	HxS	TxS	HxTxS
SPAD	2.693	732.922 *	84.791 *	9.333	4.18	58.41	23.831
Chl-*a*	3.158	2.158 *	1.589	16.52	11.150 *	7.119	7.1331 *
Chl-*b*	4.335 *	2.778	4.598	12.891	14.516	11.010	8.987 *
Car	5.112	1.889	5.115	20.150 *	12.260	9.116	7.668 *
MDA	799.322 *	4257.701 *	14,799.288 *	3119.895 *	2648.025 *	2204.624 *	232.441
APX	0.219 *	1.497 *	0.116 *	0.031 *	0.007	0.054 *	0.020 *
POX	0.020 *	0.536 *	0.265 *	0.001	0.028 *	0.031 *	0.011
SOD	0.597	28.101 *	3.135 *	0.930 *	0.037	0.967 *	0.084
Proline	0.013	0.001 *	0.078 *	0.003	0.001	0.001 *	0.001 *
Height	78,375.168 *	87,860.924 *	8455.813 *	913.530 *	1391.294 *	10,121.012 *	2547.550 *
Diameter	374.978 *	165.483 *	107.648 *	1.041	107.648 *	0.919	0.919

The data were evaluated by three-way ANOVA followed by the Tukey-HSD test at 0.05 to determine significant differences indicated by * *p* ≤ 0.05. SPAD = relative chlorophyll content, Chl-*a*: chlorophyll-*a*, Chl-*b*: chlorophyll-*b*, Car: carotenoids, MDA: malondialdehyde, APX: ascorbate peroxidase, POX: guaiacol peroxidase, SOD: superoxide dismutase, Height: plants height, Diameter: stem diameter.

**Table 2:** One-way ANOVA analysis for the three clusters created with K-mean clusters for chlorophylls (chlorophyll-*a*, and *b*), carotenoids, malondialdehyde (MDA), ascorbate peroxidase (APX), guaiacol peroxidase (POD), superoxide dismutase (SOD), and proline, df: degree of freedom.

	Cluster		Error		F	Sig.
	Mean Square	df	Mean Square	df		
Zscore (chlorophyll*a*)	24.716	2	0.394	75	62.765	0.000
Zscore (chlorophyll*b*)	2.939	2	0.948	75	3.099	0.051
Zscore (carotenoid)	7.162	2	0.814	75	8.797	0.000
Zscore (MDA)	18.332	2	0.525	75	34.932	0.000
Zscore (APX)	17.036	2	0.598	75	28.475	0.000
Zscore (POD)	16.822	2	0.577	75	29.141	0.000
Zscore (SOD)	18.260	2	0.546	75	33.471	0.000
Zscore (Proline)	7.567	2	0.844	75	8.970	0.000

**Table 3:** Correlations based on the average values for chlorophyll-*a*, and *b* (Chl-*a*, Chl-*b)*, carotenoids (Car), malondialdehyde (MDA), ascorbate peroxidase (APX), guaiacol peroxidase (POD), superoxide dismutase (SOD), and proline for all treatments combined.

	Chl-*a*	Chl-*b*	Car	MDA	APX	POX	SOD	Proline
Chl-*a*	1	ns	0.673 **	−0.679 **	−0.479 **	−0.421 **	ns	0.412 **
Chl-*b*	ns	1	ns	ns	−0.291 **	−0.291 **	ns	ns
Car	0.673 **	ns	1	−0.382 **	ns	ns	ns	ns
MDA	−0.679 **	ns	−0.382 **	1	0.562 **	0.424 **	−0.226 *	−0.691 **
APX	−0.479 **	−0.291 **	ns	0.562 **	1	0.802 **	−0.180	−0.243 *
POX	−0.421 **	−0.291 **	ns	0.424 **	0.802 **	1	−0.246 *	ns
SOD	ns	ns	ns	−0.226 *	ns	−0.246 *	1	ns
Proline	0.412 **	ns	ns	−0.691 **	−0.243 *	ns	ns	1

* correlation is significant at the 0.05 level (2-tailed) (*p* = 5%), ** correlation is significant at the 0.01 level (2-tailed) (*p* = 1%), ns: not significant.

**Table 4:** Plant height (mm) and stem diameter (mm) of corn smut infected Desszert 73 and Noa hybrids at 7 and 14 DAPI (mean ± SD, n = 5).

Plant Height					Stem Diameter			
	Desszert 73		Noa		Desszert 73		Noa	
	7 DAPI	14 DAPI	7 DAPI	14 DAPI	7 DAPI	14 DAPI	7 DAPI	14 DAPI
Control	453 ± 17.08 b	640 ± 18.26 c	544 ± 15.17 b	647 ± 5.77 c	22.92 ± 1.44 a	27.36 ± 0.77 b	25.34 ± 0.85 a	28.82 ± 0.94 b
2500	375 ± 19.15 ab	445 ± 19.15 b	450 ± 10 a	528 ± 12.58 b	26.26 ± 0.70 b	31.84 ± 0.81 c	27.88 ± 0.84 ab	33.66 ± 1.46 c
5000	345 ± 12.91 a	403 ± 15.28 ab	444 ± 23.02 a	473 ± 20.82 a	28.42 ± 0.75 b	31.94 ± 1.11 c	28.18 ± 0.95 b	34.92 ± 0.93 c
10,000	365 ± 12.91 a	383 ± 9.57 a	422 ± 13.04 a	470 ± 15.81 a	28.2 ± 0.70 b	33.94 ± 1.09 c	29.58 ± 0.68 b	35.72 ± 0.91 c

The data were evaluated by One-way ANOVA followed by the Tukey-HSD test at 0.05 to determine significant differences indicated by different letters (a, b, and c). DAPI: days after the pathogen infection.

**Table 5:** The effect of the corn smut infection on cob length (cm), cob diameter (cm), kernel weight (g), 100 grains fresh weight (g), and 100 grains dry weight (g) (mean ± SD, n = 5) of Desszert 73 and Noa hybrids at 21 DAPI.

	Desszert 73		Noa	
	Control	Infected	Control	Infected
Cob length (cm)	21.34 ±1.31 b	17.32 ± 3.33 a	23.59 ± 0.19 b	16.51 ± 0.99 a
Cob diameter (cm)	4.40 ± 0.45 b	3.62 ± 0.41 a	4.68 ± 0.16 a	3.72 ± 0.18 a
Kernel weight (g)	275.81 ± 32.07 b	161.42 ± 29.44 a	311.80 ± 24.43 b	259.30 ± 12.22 a
100 grains fresh weight (g)	42.69 ± 1.31 b	34.42 ± 3.30 a	45.46 ± 4.01 b	37.92 ± 1.04 a
100 grains dry weight (g)	9.04 ± 1.15 b	4.39 ± 0.72 a	7.99 ± 0.27 b	3.29 ± 0.15 a

The data were evaluated by one-way ANOVA followed by the Tukey-HSD test at 0.05 to determine significant differences indicated by different letters (a, b).

**Table 6:** One-way ANOVA analysis for the three clusters created with K-mean clusters for cob length, cob diameter, kernel weight, 100 grains fresh and dry weight.

	Cluster Mean Square	df	Error Mean Square	df	F	Sig.
Zscore (coblenght)	2.865	2	0.610	7	4.697	0.051
Zscore (cobdiameter)	3.170	2	0.150	7	21.077	0.001
Zscore (kernelweight)	1.650	2	0.233	7	7.067	0.021
Zscore (stograinweigt)	3.900	2	0.685	7	5.693	0.034
Zscore (stograinsusa)	4.395	2	0.032	7	136.657	0.000

**Table 7:** The effect of the corn smut infection on elements concentration (mg/kg DW) of Desszert 73 and Noa hybrids’ kernel at 21 DAPI (mean ± SD, n = 5).

	Desszert 73		Noa	
	Control	Infected Plants	Control	Infected Plants
Al	0.38 ± 0.20 a	1.07 ± 0.33 b	0.81 ± 0.08 a	1.66 ± 0.42 b
B	3.21 ± 0.02 b	2.88 ± 0.13 a	3.38 ± 0.14 a	3.00 ± 0.21 a
Ca	67.31 ± 2.64 a	75.48 ± 4.80 b	67.16 ± 1.62 a	72.60 ± 4.61 b
Cr	0.11 ± 0.003 a	0.11 ± 0.008 a	0.12 ± 0.01 a	0.14 ± 0.005 a
Cu	2.85 ± 0.15 a	2.55 ± 0.21 a	2.34 ± 0.09 a	2.46 ± 0.14 a
Fe	12.34 ± 0.26 a	12.12 ± 0.51 a	12.59 ± 0.48 a	13.37 ± 0.75a
K	6132.94 ± 77.68 a	6093.44 ± 113.27 a	5966.32 ± 202.62 b	5716.64 ± 148.06 a
Mg	789.62 ± 16.81 b	686.91 ± 7.66 a	746.868 ± 12.12 b	690.20 ± 10.74 a
Mn	9.41 ± 0.40 b	7.52 ± 0.52 a	8.45 ± 0.40 b	7.34 ± 0.53 a
Na	21.35 ± 1.35 a	22.65 ± 0.84 a	21.73 ± 1.03 a	28.05 ± 1.96 b
P	2998.66 ± 151.78 b	2687.77 ± 63.30 a	2777.86 ± 62.22 a	2677.32 ± 50.32 a
Pb	0.11 ± 0.003 a	0.18 ± 0.006 b	0.13 ± 0.023 a	0.13 ± 0.007 a
S	870.61 ± 42.84 a	930.92 ± 54.18 b	824.195 ± 18.56 a	940.99 ± 64.51 b
Sr	0.62 ± 0.09 a	0.79 ± 0.04 a	0.56 ± 0.03 a	0.47 ± 0.11 a
Zn	28.38 ± 0.25 b	26.86 ± 1.94 a	24.35 ± 0.90 a	23.71 ± 1.54 a

The data were evaluated by one-way ANOVA followed by Tukey-HSD test at 0.05 to determine significant differences indicated by different letters (a, and b). Al: Aluminum, B: Boron, Ca: Calcium, Cr: Chromium, Cu: Copper, Fe: Iron, K: Potassium, Mg: Magnesium, Mn: Manganese, Na: Sodium, P: Phosphorus, Pb: Lead, S: Sulfur, Sr: Strontium, and Zn: Zinc. DAPI: days after the infection, DW: dry weight.

## Data Availability

Data are contained within the article.
